# Supplementary material for: Energy-harnessing problem solving of primordial life: Modeling the emergence of catalytic host-nested parasite life cycles
Source: PLoS One. 2023 Mar 27;18(3):e0281661. doi: 10.1371/journal.pone.0281661 (PMC10042343; doi:10.1371/journal.pone.0281661)
Supplement: S1 Fig — Parasite growth is unlimited (bacteria, phage), as long as the habitat nourishes it. Once the host loses the ability to nourish the parasites, they are condemned to perish. The association of a dedicated hyperparasite (phage) with a given parasite (bacteria) forming nested parasite pairs is a very effective and robust taming strategy for a parasitized host (both populations fluctuate around their equilibria). (DOCX) [file pone.0281661.s001.docx]

**S1 Fig 1**
