# Supplementary material for: Energy-harnessing problem solving of primordial life: Modeling the emergence of catalytic host-nested parasite life cycles
Source: PLoS One. 2023 Mar 27;18(3):e0281661. doi: 10.1371/journal.pone.0281661 (PMC10042343; doi:10.1371/journal.pone.0281661)
Supplement: S2 Fig — An initially stabilized host-nested parasite unit recruits symbionts (red) that catalyze energy transformation. Degenerate endosymbionts are replaced by fresh symbionts (green) that catalyze the next transformative round. A trio of such host-nested parasite units and two energies are required for adequate system stabilization and to build a catalytic life cycle. (DOCX) [file pone.0281661.s002.docx]

**S1 Fig 2**
