# Supplementary material for: Energy-harnessing problem solving of primordial life: Modeling the emergence of catalytic host-nested parasite life cycles
Source: PLoS One. 2023 Mar 27;18(3):e0281661. doi: 10.1371/journal.pone.0281661 (PMC10042343; doi:10.1371/journal.pone.0281661)
Supplement: Attachment — Submitted filename: Response to Reviewers.docx [file pone.0281661.s003.docx]

**Response to Reviewers manuscript PONE-D-21-05736**

**Reviewer #1:** The authors use an energy-based framework to investigate the conditions required for a LUCA-like organism and its parasite to survive. They consider hyperparasite populations (where a parasite is itself infected with another parasite, e.g., a prokaryote and a phage). They suggest that fitness between host and parasite are linked, due to the Lotka-Volterra equations they use to model the system. Using the equilibria from the Lotka-Volterra model they substitute these into ODEs defining the total energy in the system and use this to define the stability of the system. They also discuss a framework for “evolutionary problem solving”. I liked the narrative presented in the introduction – it gave a good introduction to the work. However, I think a little more justification of how this study fits into the research area is necessary.

I have concerns about the methodology and models used. Firstly, the authors choose to model prokaryote-phage dynamics with Lotka-Volterra equations, without justification for why this is appropriate. It is incredibly unlikely that prokaryote-phage dynamics are so simple as to be suitably modelled by Lotka-Volterra equations; for example, Lotka-Volterra equations ignore behaviours like competition between different phages. If Lotka-Volterra models are indeed sufficient to model the dynamics, then I felt that as a reader I needed a bit more persuading.

The *Reviewer* rightly points out that we had not properly introduced the background, as to why we are convinced that Lotka-Volterra (LV) models are not only appropriate, but the most appro-priate framework available for this particular setting.

We now provide a short introduction and lay out our reasoning.

First and foremost, in a comparative analysis T. Royama convincingly argues that the LV equa-tions cover the widest scope; in other words, these equations are the most general [1] (e.g. p.80).

Second, the *Reviewer* mentions that for instance competition between different phages is not accounted for. We now argue that intra- and interspecies competition is one of strong features of these equations that has extensively been modelled. Indeed, Campbell’s model is reflecting intraspecific competition, formally represented by a habitat restriction term$\left( 1-\frac{X}{L} \right)$ in the equation (*X* being the host, *Y* the phage) [2]:

$$\frac{dX}{dt}=X(a_{1}\left( 1-\frac{X}{L} \right)-a_{2}Y)$$

$$\frac{dY}{dt}=Y\left( -b_{1}+b_{2}X \right)$$

Campbell also expands this model to interspecific competition, arguing that based on the *com-petitive exclusion principle* the consideration can be limited to the fittest host: “Reasoning in this way, one can conclude that a phage can survive without destroying its host simply by having chosen: the best-adapted from a group of competing hosts” [2]. We now expanded the original LV-equations to Campbell’s model including intraspecific competition (new Fig 1-3).

More formally, *the competitive exclusion principle* authored by J. Grinnell (1904) and G. F. Gause (1934), and popularized by G. Hardin (1960) stipulates that two “species survive inde-finitely only when they occupy different niches in the microcosm in which they have an advan-tage over their competitors” [3-5].

Third, empirical data show that these models accurately reflect for instance bacteriophage inter-actions in aquatic systems [6]; moreover, oceanic and lake virioplankton dynamics are also consistent with LV [7-8].

Collectively, the LV equations have extensively been used to model bacteria-phage interact-tions, including competition, and natural population dynamics are consistent with it. The LV-framework expanded to include Campbell’s model therefore represents an adequate instrument for our own model. In terms of competition, we only model one, the fittest host population per phage, not expanding the model into more detailed intra-specific competition.

Second, the first paragraph on page 5 is described as a proof and attempts to justify linking the fitness between the host and the prokaryote-virus pair. I would first argue that this is not an actual proof and should not be described as one; instead, it is a verbal argument.

We now have included extended elements of a formal proof based on Campbell’s model exten-ded to a hyperparasite framework (Methods).

Next, more crucially, because of the choice of LV equations, the equilibrium populations of the prokaryote and virus are only dependent on the resources of their environment (which in this case is the host). They then suggest that because of this, host fitness can be synchronised with the fitness of the prokaryote and virus. However, I fear this leads to a logic error – just because host size implies prokaryote-virus size, the reverse is not necessarily implied; and hence the fitness of hosts is not necessarily dependent on the fitness of the prokaryote or virus.

We addressed this issue and indeed show that our initial reasoning is valid. This is illustrated by a mechanism we call *fitness synchronization* using Campbell’s model expanded to a hyper-parasite framework (Methods).

I don’t agree with how the authors have modelled the virulence in the model. They first suggest that virulence can take values between 0 (avirulent) and 1 (maximal virulence) on page 11, which seems reasonable. However, later they model the virulence on pages 12 and 15 with a step-function, where the virulence can only be avirulent or maximally virulent, dependent on the energies in the system. It seems unrealistic that the virulence is modelled with a step function as one would expect virulence to be able to vary between being avirulent and maximally virulent.

As suggested by the *Reviewer*, we have now expanded the original stepwise function to the con-tinuous model and show that our conclusions still are valid (new Fig 1B).

I was confused about the labelling of populations and why they have chosen them to interact in the way they do. For example, on pages 14 and 15, it was very unclear to me why the virulence of the pair (X1,Y1) was dependent on the active energy source E1, but the virulence of pairs (X2,Y2) and (X3,Y3) are dependent on the energy store E2.

We acknowledge that these arguments merit a more exhaustive development and have added a new narrative at the end of the *Introduction* section.

Furthermore, when they define how E1 and E2 vary with time on page 15, dE1/dt is not dependent on X1, and dE2/dt is not dependent on X2, or Y2. I presume there is reasoning for these choices; however, as a reader, the reasons for these choices were left very unclear.

It is irrelevant which of the prokaryote-phage pairs (*X, Y*) catalyze new Energy *E*. Other possible realizations would be that both *X* and *Y* catalyze *E*, or alternatively only *X* (see as already stated in L357).

Finally, I found the figures very hard to interpret. None of the figures had axis labels, and without reading the paper it is very unintuitive to have virulence and energy being plotted on the same set of axes. As you go from figures 1-3b the complexity of the figures increase. By figure 3b it had become uninterpretable. There are far too many things being plotted on one set of axes (particularly in figure 3b) and this meant that I was unable to understand what I was supposed to intuit from the figure. This led to a disconnect between the results section and the figures, where I was unsure how they obtained their conclusions. The figures need to be simplified in order to emphasise the results and narrative that the authors want the reader to understand. Likewise, I think the authors should highlight how they get their results from the figures in the results section to make it clearer.

As suggested we simplified the Figures, added axis labeling and overall improved readability of the graphical material.

**Reviewer #2:** The manuscript presents a model of the dynamics of a host, its parasite and a hyper-parasite (nominally some simple host organism, a bacteria and a bacteriaphage). They use a Lotka-Volterra model framework to study how this interaction depends on 'energy' constraints of the host and virulence of the parasite. The dynamics of the host are not explicitly modelled, only those of the two parasites and the 'energy'. They show that simple systems can be maintained, but that a system of 3 host-parasite units and 2 forms of energy gives more reliable long-term persistence.

The manuscript was interesting, tailored to a topic of early life-forms that I'd not really thought about before, but I found it pretty confusing. A few reasons:

I don't know if this is a journal requirement but I would strongly recommend moving the methods to before the results. It is close to impossible to interpret the results of a model without first knowing what the model assumes. In this case I spent most of my first read thinking you were looking at a completely different (and very wrong) model because the model stated at the start of the results is both the simplest possible model and actually quite misleading (because you assume b1,b2<0 and that is not made obvious).

As suggested by the *Reviewer* we now have reorganized the manuscript and moved the *Methods* before the *Results* section.

I'm not sure why you chose a Lotka-Volterra model. While I became a bit less worried once I discovered your assumptions on b1 and b2, it still seems odd not to use the more classic epidemiological models (e.g. SIR-type frameworks) which are expressly designed for infectious disease systems. Why use what is more of a competition/predator-prey model? Perhaps an argument can be made for this being more suitable, but if so why choose the version of it with no intra-specific competition, meaning you get exponential growth in the absence of the hyper-parasite?

We intended to treat host and parasite equally, for that reason we allowed the parameters to become negative. All other things equal, this merely is a matter of representation. We addressed this issue and modified the expanded Campbell model that now uses positive value terms as suggested by the *Reviewer*.

Moreover, the *Reviewer* rightly points out that we had not properly introduced the background, as to why we are convinced that LV models are not only appropriate, but the most appropriate framework available for this particular setting.

We now provide a short introduction and lay out our reasoning.

First and foremost, in a comparative analysis T. Royama convincingly argues that the LV equations cover the widest scope; in other words, these equations are the most general [1] (e.g. p.80).

Second, the *Reviewer* mentions that for instance competition between different phages is not accounted for. We now argue that intra- and interspecies competition is one of strong features of these equations that has extensively been modelled. Indeed, Campbell’s model is reflecting intraspecific competition, formally represented by a habitat restriction term$\left( 1-\frac{X}{L} \right)$ in the equation (*X* being the host, *Y* the phage) [2]:

$$\frac{dX}{dt}=X(a_{1}\left( 1-\frac{X}{L} \right)-a_{2}Y)$$

$$\frac{dY}{dt}=Y\left( -b_{1}+b_{2}X \right)$$

Campbell also expands this model to interspecific competition, arguing that based on the *competitive exclusion principle* the consideration can be limited to the fittest host: “Reasoning in this way, one can conclude that a phage can survive without destroying its host simply by having chosen: the best-adapted from a group of competing hosts” [2]. We now expanded the original LV-equations to Campbell’s model including intraspecific competition (new Figs. 1-3).

More formally, *the competitive exclusion principle* authored by J. Grinnell (1904) and G. F. Gause (1934), and popularized by G. Hardin (1960) stipulates that two “species survive indefinitely only when they occupy different niches in the microcosm in which they have an advantage over their competitors” [3-5].

Third, empirical data show that these models accurately reflect for instance bacteriophage interactions in aquatic systems [6]; moreover, oceanic and lake virioplankton dynamics are also consistent with LV [7, 8].

Collectively, the LV equations have extensively been used to model bacteria-phage interact-tions, including competition, and natural population dynamics are consistent with it. The LV-framework expanded to include Campbell’s model therefore represents an adequate instrument for our own model. In terms of competition, we only model one, the fittest host population per phage, not expanding the model into more detailed intra-specific competition.

Some of the language was difficult to interpret. You talk a lot about the equilibria and stability, but I'm not convinced that is really what you mean (the classic L-V model you use does have an equilibria but it is unstable).

As suggested by the *Reviewer* we added a narrative, both in the *Introduction, Materials and Methods* and *Discussion* sections to clarify the meaning of these terms.

Briefly, host-nested parasite interactions generally imply large fluctuations without necessarily achieving equilibrium states with diminishing amplitudes. Therefore, our model is a first step allowing a living system to continue evolving, however, still with life-threating fluctuations. This is an intermediate step that paves the way for more elaborate next moves that imply syner-gies between hosts and parasites.

In the revised manuscript using the expanded Campbell model, intraspecific competition leads to a smoothening of these fluctuations (new Fig 2B).

I also struggled to fully understand what is claimed to be the main result. You claim that a 3-unit system is capable of persisting in the long-term, which does appear to be true, but your simple system (i.e. figure 1) also appears to persist, so why is that not so important?

While the first host-nested parasite unit provides an initial stability, it still is vulnerable to inter-nal and external challenges. For this reason, further evolution of the system clearly is critical and this is brought about by synergistic host-nested parasite interactions (see above).

In specific terms, in such a critical situation the three-unit system can mobilize stored energy *E2* and transform it into operating power *E1*, a process that buys time for the system to resolve the critical situation.

Further detailed comments are below.

P3 L65 - I don't quite follow the reasoning for why the parasite must itself be parastised. Please could you elaborate.

We argue first and most importantly on a mechanistic basis; one can posit that replication is a core requirement for living systems, and the process of replication inherently is imperfect, which generates molecular parasites of subgenomic size [9]. As a corollary, these parasites fol-low a simple set of rules that govern their destiny; they are smaller, therefore much more abun-dant than the parent molecule, creating a favorable probability space for further molecular evo-lution and a pervasive occupation of all available niches. This was particularly true in early life and has since been compensated for by a variety of mechanisms, particularly proofread and repair activity.

Secondly, based on empirical data, one is tempted to accept that this is what happens in real life. Indeed, all parasites naturally undergo parasitism, a nested chain process that ends only when parasites degenerate too far (which can be the case for endoparasites), and only then they’ll face evolutionary dead-ends that become inevitable [10].

P3 L72 - The cyclic pattern of classic L-V dynamics only occur if there is no intra-specific competition, and are structurally unstable. In the more general case there may be damped oscillations to a coexistence equilibrium. I think it is a bit strong to just state that L-V dynamics give cycles without the caveats.

As stated above, we now modelled nested parasitism using modified Campbell equations and go on to show that populations are likely to persist under these conditions (new Fig 1-3).

P4 L86 - Can you clarify what you mean by 'the phage population Y stabilizes X in its host'? That it causes X to have a stable equilibrium/cycle that it would not otherwaise have? Or something else?

We would argue that this is a primitive stabilizing mechanism that is easier at hand in primordial life settings than more labor-intensive, sophisticated alternatives, such as defense and immu-nity.

P4 L92 - With reference to the point above, I think the assumption of this simple L-V dynamic needs justification. It is also extremely important that one has read the methods section to discover that you assume b1,b2<0. On first read I assumed these were positive (because if they weren't, why not just use a minus sign?) and then the Y equation made absolutely no sense as a representation of a parasite.

We intended to treat host and parasite equally, for that reason we allowed the parameters to become negative. All other things equal, this merely is a matter of representation. We addressed this issue and modified the expanded Campbell model that now uses positive value terms as suggested by the *Reviewer*.

There are still questions though. Without the phage the bacteria grows exponentially (as you state below). While that feels more reasonable (because in this case the bacteria is no longer being parasitised) there is a question of whether a host could really harbour an infinite number of bacteria. It therefore seems questionable to me how suitable these equations are. Why did you not just use an SIR-type model that is expressly designed for infectious disease systems?

We acknowledge that SIR models would be a valuable alternative and appreciate the role they have played in the recent pandemics; this did not escape our attention, and we are aware that the model was initiated in our country by Bernoulli in 1760, and later popularized by Kermack and McKendrick [11]. As mentioned before, for a number of reasons we considered the LV framework more suitable; i) the fact that the LV equations are the most generally valid for host-parasite interactions, ii) they have extensively been used to model bacteria-phage interactions including competition, and finally, iii) natural bacteria-phage populations evolve consistent with LV-dynamics. These arguments have now been incorporated in the text section.

P4 L95 - It is true that the equilibria are independent of the ICs, but the cycle the populations experience is not. This is actually an important point as it is not true of most systems of ODES generically.

In the revised manuscript we show using the Campbell extension that the oscillations smooth out due to the intraspecific competition for all ICs (new Fig 2, new Fig 3).

P5 L104 - By the 'stabilized equilibria populations' I assume you mean the equilibria stated just above. But these are not the values that the populations will ever take (unless they happen to have ICs at precisely those values). So why would they be in the fitness.

This issue is now specifically addressed by the expanded Campbell since the fluctuations are smoothed out due to the interspecific competition.

Moreover, I'm unclear about the definition of 'size of habitat'. That seems different to me to 'the number of possible encouters between members of X and Y'. Do you mean a couple of the parameters specifically?

We now have clarified this issue using the extended Campbell-Model (new Fig 1-3).

P5 L109 - Up to now I had taken it as an assumption that the host population size was fixed. Given that the bacteria growth rate does not depend on the host size I assumed that must be the case. However, it now appears that you assume that the host population size is variable (I guess it must be for fitness to mean anything). So why does bacteria growth not depend on host population size?

It actually does, which is discussed using the modified Campbell’s equations and developed under the heading of synchronization (Methods). We surmise that this issue is now appro-priately addressed.

P5 L113 - Isn't the growth rate of an equilibrium population precisely 0? I assume you are meaning the cycles which the populations are on, but I would personally not define that as an equilibrium.

As long as the replication dynamics of the host is slower than the replications dynamics of the nested parasite pair, then the equilibria of the parasite and hyperparasite can slowly adapt to the slower growth rate of the host (see above explanations on synchronization and Methods).

P5 L116 - I think the statement about reaching far beyond other models is a stretch. Plenty of models have looked at dynamics of host-parasite-predator or host-parasite-parasite, or even nested and network models. I'm not saying you've not done anything novel, I just think you need to be carteful how miuch you claim.

We now added a *Discussion* section on the limits of our model.

P6 L124 - It'd be helpful to state from the off what you mean by 'virulence'. The term has different meanings in different fields. I think here it is about infection?

We defined it in the original model as being 0 or 1, and now as a number between 0 and 1 (Methods).

P6 L137 - Why did you choose this 'on/off' virulence rather than a continuous function?

As suggested we now modelled a continuous function via a number between 0 and 1 (new Fig 1B). But since the dynamics does not depend on the continuity of the virulence function, we still used an on/off virulence for our simulations in the revised model.

P6 L142 - WHat do you mean by 'homeostasis'? Just that everything persists? It's not obviously on a repeating cycle from the plot in figure 2.

Homeostasis here is the fact that the energy level is being held constant in relation to the number of hosts. We have added this clarification to the main text body.

P6 L144 - I'm confused as to what Energy is. It sounds as if it must be some property of the host? But you say it's something to do with genetic drift and environmental change? Why does host energy suddenly start dropping in the figure - are you assuming some external change that has caused it or is there something in the model?

We now added an introductory narrative on Energy, Energy forms and their role.

Energy drops in any event if nothing happens in the host-parasite habitat. For this reason, new catalytic host-parasite pairs need to be recruited to compensate for that loss.

Also, the figure legends contain too little information. Please state what each line represents and the parameter values used. Also, please consider a different colour combination as it is hard for colourblind readers to differentiate the lines.

We now improved the Figure legends and the graphical representations.

P6 L146 - What do you mean by it being 'considerably unstable'?

By “considerably unstable” we mean subject to strong external fluctuations and drift (inevitable degenerative process leading to breakdown of E1 catalysis). We have added this clarification to the main text body.

P8 L176 - So is this larger model demonstrably more robust/stable? The dynamics in figs 3a/b look no more or less stable compared to figure 2 on first look.

We now visualized the differences in a dedicated Figure (new Fig 3).

P10 L245 - Why does sum(P_j)=e^T P? What is e^T?

We now included the formal definitions that you will find below in the *Methods* section.

The column-vector $e_{i}=(0,0,..,1,0,\ldots,0)^{T}$ denotes the $i_{th}$ basis vectors of n-space with entry one at position$i$ and zero else. The column-vector $e=(1,1,.\ldots,1)^{T}$ is the n-vector with all entries one. $e$ is sometimes called the summation vector since it sums up all the entries of the vector it is multiplied with: $e^{T}x=\sum_{i} x_{i}$. If $p$ is a probability distribution vector, then $p_{i}\geq0$ for all $i$ and $e^{T}p=1$. We then write for the expected value of n-vectors $x,y$: $E\left( x \right)=\bar{x}=x^{T}p=e^{T}diag\left( x \right) p \mathrm{and}$ $E\left( y \right)=\bar{y}=y^{T}p respectively,$ and for their variance and covariance:$var\left( x \right)=\bar{x^{2}}{-\bar{x}^{2}=e}^{T}diag(x)^{2}p-\bar{x}^{2}$, $cov\left( x,y \right)=\bar{xy}-\bar{x}\bar{y}=e^{T}diag\left( x \right)diag\left( y \right)p-\bar{x}\bar{y}$.

P11 L260 - How exactly does virulence impact the host? You have this approach of implicitly assuming host dynamics are linked to the parasite and hyper-parasite, but the equations you've described do not include the virulence term. (SEE BELOW)

P12 L275 - This is another example of things coming in the wrong order. We are now shown an updated model that does include virulence. This is key, as up to now there has been no sign at all that v was ever impacting anything. However I'm still a bit unclear which parasite is being virulent. v=0 implies the bacteria's fitness does not depend on the phage, implying that it is the phage that is capable of being virulent. But everything I'd read up to now led me to believe it was a question of the bacteria being virulent to the host.

Our model stipulates that the energy level *E1* drives virulence of the bacteria-phage pair *X1, Y1*. When it is high, virulence is low, and conversely, when *E1* is low, virulence is high.

P12 L279 - This ODE for energy implies that in the absence of the phage energy is strictly decreasing, but that is not what is shown in figure 1. Why is that?

As stated above: “Energy drops in any event if nothing happens in the host-parasite habitat. For this reason, new catalytic host-parasite pairs need to be recruited to compensate for that loss”. This is now addressed in the main text body and Fig 2B.

P14 L327 - I don't follow whether this procedure is somehow embedded in the model. I think it must be for the point above about how the energy increases in figure 1 to be possible. You explain it for the larger model below here, but not for the simple case.

We addressed this in the previous section.

Discussion - This is very brief. I'd particularly like to see you draw parallels from your work to existing models of host-parasite-hyper-parasite that exist. It might also be a place to comment on your choice of model framework and why it would be suitable in this case.

We now discussed existing host-nested parasite models in the Introduction, and expanded the Discussion section to highlight differences with our model.

**References**

1. Royama T (1971) A comparative study of models for predation and parasitism. Population Ecology 13: 1–91.
2. Campbell A (1961) Conditions for the existence of bacteriophage. Evolution 15: 153–165.
3. Hardin G (1960) The Competitive Exclusion Principle. Science 131;1292-1297.
4. Grinnell J (1904) The Origin and Distribution of the Chest-Nut-Backed Chickadee

The Auk 21:364-382.

5. Gause GF (1934) The struggle for existence. The Williams and Wilkins Co., Baltimore, Md.
6. Thingstad TF (2000) Elements of a theory for the mechanisms controlling abundance, diversity, and biogeochemical role of lytic bacterial viruses in aquatic systems. Limnol Oceanogr 45: 1320–1328.
7. Hennes KP, Meinhard S (1995) Significance of Bacteriophages for Controlling Bacterioplankton Growth in a Mesotrophic Lake. Appl Environment Microbiol 61: 333–340.
8. Parsons RJ, Breitbart M, Lomas MW, Carlson CA (2012) Ocean time-series reveals recurring seasonal patterns of virioplankton dynamics in the northwestern Sargasso Sea. The ISME Journal 6: 273–284.
9. Chen I (2015) Replicating towards complexity. Nature Chem 7:191–192.
10. Newton ILG, Borderstein SR (2011) Correlations Between Bacterial Ecology and Mobile DNA. Curr Microbiol 62:198–208.
11. Kermack W, McKendrick A (1927) A contribution to the mathematical theory of epidemics. Proc R Soc London A 115:700-721.
