# Supplementary material for: Energy-harnessing problem solving of primordial life: Modeling the emergence of catalytic host-nested parasite life cycles
Source: PLoS One. 2023 Mar 27;18(3):e0281661. doi: 10.1371/journal.pone.0281661 (PMC10042343; doi:10.1371/journal.pone.0281661)
Supplement: Attachment — Submitted filename: Response to Reviewer.docx [file pone.0281661.s004.docx]

Rebuttal letter for PONE-D-22-29899

Energy-harnessing problem solving of primordial life: modelling the emergence of catalytic host-nested parasite life cycles

**Reviewer #1: Review report of PONE-D-22-29899**
Energy-harnessing problem solving of primordial life: modelling the emergence of catalytic host-nested parasite life cycles by Bernard Conrad, Christian Iseli, and Magnus Pirovino.

The paper presents a model of the energetic, replicative conditions of LUCA-like organisms and their parasites using Lotka-Volterra equations adapted to a nested parasite pair.

In my view, the paper could be published. Based on the stated assumptions, the model displays, by and large, the [damping oscillatory] behavior I expect.

So I am not questioning the work as such, only its relevance. I don’t think the LV model correctly presents energetics, i.e., thermodynamics, that drives evolution. We can do better. My point is substantiated by Mäkelä T, Annila A. Natural patterns of energy dispersal. Phys. Life Rev. 2010 7, 477-498. doi:10.1016/j.plrev.2010.10.001.

Sure, the Authors refer to the assumptions of the LV model, but still, in my view, the Readers would benefit from knowing that the thermodynamics of open systems describes abiogenesis without assuming LUCA, the hereditary material, etc. I am not insisting on this matter, only pointing out that mathematical modeling is not advancing our understanding of evolution, perhaps only obscuring it with model concepts and parameters that cannot be unambiguously related to the substance in evolution. For example, homeostasis is fine by corresponding to thermodynamic balance, while correspondence of the parasite capacity of the hosts in a given habitat to thermodynamics is vague.

Of course, one could argue that modeling is necessary to reduce the complexity to something practical, but in my view, not at the cost of comprehension. In thermodynamic terms, the oscillatory behavior results from free energy consumption that is large compared to the energy bound in populations. No question, the Authors’ model displays some conceivable scenarios, but it does not explain why things happen, causes and consequences, i.e., forces and changes in motion.

As suggested by the Reviewer #1, we now fully acknowledge that a growing number of models were formulated in recent years aiming to apply thermodynamics to the origin of life and evolution, and dis-cuss the respective ramifications and implications (new refs. 49-51; swap of refs. 31 and 32).

Specifically, we added a new Materials and Methods section describing the *catalytic principle* and high-lighted its role in our model and relationship with the cornerstone second law of thermodynamics.

In addition, at the end of the discussion session we enumerate a number of recent models applying thermodynamics to this field. Specifically, as suggested by Reviewer# 1, we mention that statistical physics of open systems were developed without assuming LUCA (ref. 51). We furthermore also high-light how such open systems reframe the traditional views of enzymes (ref. 51), a concept that is cen-tral to the *catalytic principle*.

In sum, we are convinced to have fully addressed the issues raised by Reviewer# 1.
